# Vulnerability and risk assessment of the coexistence of oil and fishing industries in the Southern Gulf of Mexico

**DOI:** 10.1007/s10661-026-15583-9

**Published:** 2026-06-24

**Authors:** Eduardo Cuevas, Alejandro Espinoza-Tenorio, Guadalupe Díaz-Gutiérrez, Raúl E. Lara-Mendoza, Ricardo Cavieses-Núñez, Jorge A. Trujillo-Córdova, Johnny B. Cruz-Pech, Deysi G. Cupido-Santamaría, Gerardo Peña-Mis, Dora R. Ramos-Muñoz, Abigail Uribe-Martínez

**Affiliations:** 1 Universidad Autónoma de Baja California, Ensenada, México; 2 El Colegio de La Frontera Sur, Unidad Campeche, Lerma, México; 3 Instituto Mexicano de Investigación en Pesca y Acuacultura Sustentables, Secretaría de Agricultura y Desarrollo Rural, CDMX, México; 4 Departamento Académico de Ingeniería en Pesquerías, Universidad Autónoma de Baja California Sur, La Paz, México

**Keywords:** Marine spatial planning, Sensitive areas, Spatial analysis, Potential interactions, Oil spills, Maritime seascapes’ governance

## Abstract

In the southern Gulf of Mexico, the oil and fishing industries coexist in a complex, historically unbalanced relationship that remains insufficiently documented. Constructing spatially explicit knowledge about these interactions is essential to inform decision-making, reduce conflicts among regional stakeholders and in the end, benefit the parties through marine territorial planning that enables stronger governance elements for a socioenvironmental responsible coexistence in the seascape. This study assessed the spatial overlap between the space used by industrial and small-scale fishing fleets and oil industry-related operations—including vessel traffic, marine infrastructure, and oil spills—through quantitative vulnerability and risk analyses. We integrated multi-source spatial data to evaluate both individual and cumulative vulnerabilities, as well as the risk of interactions between fishing fleets’ space use and surface oil presence. Results highlighted two high-vulnerable and high-risk zones: (1) the eastern coast of Tabasco and western coast of Campeche, where small-scale fishing fleets face constant exposure to large vessels, oil platforms, and pipelines; and (2) industrial fishing fleets at the north of Ciudad del Carmen, which showed a higher probability of encountering surface oil. These findings represent a significant contribution to the understanding of risk in maritime spaces where small-scale fisheries and a recently declared marine protected area coexist with the oil industry. Our framework offers a foundation for integrated territorial management that seeks to reduce conflicts between oil extraction and fishing activities, while supporting broader conservation goals in one of Mexico’s most ecologically and economically important marine regions.

**Supplementary Information:**

The online version contains supplementary material available at 10.1007/s10661-026-15583-9.

## Introduction

One of the biggest challenges in managing seascapes is the harmonization of coexisting extractive activities and the need to preserve essential habitats for sustaining the viability of the oceans in the long term. Trade-offs between different uses of territory need to be evaluated and consensual for minimizing the social and ecological conflicts. This scenario has been the case of the energy and mining industries that interact with the livelihoods of coastal communities, which frequently rely on fisheries and tourism services, making them susceptible to impacts from the former industries (Wakida-Kusunoki & Caballero-Chávez, 2009; Quist & Nygren, 2015; Kusters et al., 2025). Also, piracy (mostly outboard engines robbery in open seas) in those maritime seascapes adds more complexity to the marine territorial use in a secure manner, highlighting the need for harmonized territory planning, surveillance, and fostering the right for a secure employment in both the oil and fishing industries (Ramos-Muñoz et al., 2019).

Ranked within the top 20 oil-producing nations in the world, the oil industry in Mexico historically contributed as an essential percentage of the gross domestic product. However, in the last decade, its proficiency has depleted, and its contribution has been significantly reduced, while the associated socioenvironmental conflicts remained (Chisadza et al., 2024; Ferrari et al., 2024).

Small-scale fisheries are some of the most vulnerable economic activities in the disputed areas (Andrews et al., 2021), and they are often marginalized in sea governance, which leads to spatial constraints that hinder their development (Coronado et al., 2024). Generally, most small-scale and industrial fishing activity is concentrated in waters less than 200 m deep, and those areas have also increasingly been occupied by offshore energy-producing infrastructure, responsible for producing nearly 30% of the world’s oil (Paolo et al., 2024). The spatial overlap between the oil industry and fisheries results in a series of frequent and interconnected impacts, including the displacement of fishers from traditional fishing areas due to increasing coastal traffic and infrastructure, as well as the catastrophic consequences of oil spills on the fishing industry (Pascoe & Innes, 2018).

In this context, a spatiotemporal assessment of the coexistence between the fishing and oil industries is essential for implementing timely management approaches such as marine spatial planning and ecosystem-based management (Baltaoglu, 2025; Holsman et al., 2017; Stelzenmüller et al., 2010). The spatial analysis tools have proven to facilitate integral assessments of the territorial coexistence by multiple claims, as they synthesize the geographic configuration of the conflict and facilitate the assemble of expert knowledge with quantitative data. This approach also enables the assessment of the social and ecological vulnerability and risk to the oil industry activities and the presence of oil, both individually and cumulatively for the components of the seascape (Nelson et al., 2015; Façanha-Camara et al., 2021).

In the southern Gulf of Mexico (sGoM), the impacts of the domestic offshore oil industry include the environmental pollution by spills of distinct magnitude (Uribe-Martínez et al., 2024), which affect the fishing resources, as well as the fishing gears and boats, and those impacts jeopardize the coastal communities’ welfare because of the environmental degradation (García-Cuellar et al., 2004).

In sGoM, the small-scale and the industrial fishing fleets comprised approximately 5,362 and 258 vessels, respectively, in 2010 (Nava-Fuentes et al., 2018). The former fleet primarily uses small fiberglass boats (6–8 m), and most of their catches come from shallow waters, where they target multiple species for human consumption, supporting local and regional markets, and significantly contributing to food security (Montejo-Damián et al., 2022). The industrial fleet includes vessels (12–24 m) made of wood, iron, or fiberglass hulls that may use different fishing gear to primarily capture species of the Serranidae, Lutjanidae, and Sparidae families (Quijano et al., 2023). The offshore shrimp fleet includes trawlers that target pink (*Farfantepenaeus duorarum*), white (*Litopenaeus setiferus*), and brown shrimps (*Penaeus aztecus*) in the sGoM.

In 2019 it was decreed a 500-m security buffer around oil wells, platforms, and other hydrocarbon extraction facilities, as well as 2500 m around any maritime hydrocarbon exporting point such as the Single Point Moorings (SPM) (Diario Oficial de la Federación, 2019), which bans public access to those called security zones. That strategy increased the conflict of the oil industry with the small-scale and industrial fisheries, which have claimed the use of their historic marine territory now occupied by the oil industry. Nowadays, the local fishermen must do longer trips, implying higher operation costs, higher exposure to piracy, and increasing risk because of the inadequacy of the small boats for ocean trips (Quist & Nygren, 2015; Ramos-Muñoz et al., 2019; Salazar-De-la-Cruz et al., 2020).

### Vulnerability and risk in marine spatial management

Vulnerability is defined as the result of exposing a sensitive component of a system to multiple pressures while also considering its intrinsic and extrinsic conditions to cope with the impact (Cuevas et al., 2019; Zacharias & Gregr, 2005). In these contexts, we define a threat as a process, event, or condition that has the potential or probability to cause damage or impact but is not necessarily occurring at present; there is a probability that it may occur and generate adverse impacts if it materializes. At the same time, pressure is a factor or action that is currently exerting a negative influence or impact on a system, and it is related to what is already occurring. It pushes the system toward an altered state, in a similar approach to Aarflot et al. (2024) and Pirasteh et al. (2024).

In a similar context, from a mostly engineering perspective, Kaynia et al. (2008) defined risk as the measurement of the likelihood and severity of an impact on life, health, property, or the environment, a definition also accepted in marine risk assessments and management (Gibbs & Browman, 2015; Stelzenmüller et al., 2015). Holsman et al. (2017) defined risk as quantifying the likelihood of adverse events occurring, plus their consequences when they occur. Mathematically speaking, risk quantification has been proposed as the multiplication of the vulnerability of the subject by the probability that a threat occurs (Eidsvig et al., 2011).

The selection of the best methodological approach for vulnerability and risk assessments will depend on the best available data and its nature. Assembling different approaches is a feasible resource for contributing the best information for well-informed knowledge and criteria, as well as to build more robust assessment strategies for multiple data availability and the seascape’s conditions (Holsman et al., 2017; Tyack et al., 2022).

In this context, spatially explicit expert information becomes a key element for completing the evaluation of spatial interaction risk between two of the most critical extractive industries, as is the case in the sGoM. The assemblage of traditional ecological knowledge and systematically acquired quantitative data has been successfully used in other vulnerability (Camara et al., 2021; Cuevas et al., 2019; Nelson et al., 2015) and risk assessments, as well as for management analysis (Bethel et al., 2014, 2022).

Vulnerability and risk to anthropic and natural pressures and threats have been extensively evaluated from multiple numerical and spatial approaches (Bethel et al., 2022; Cruz-Ramírez et al., 2024; Gibbs & Browman, 2015; Helle et al., 2020; Stelzenmüller et al., 2015; Tyack et al., 2022). The ultimate objective of all those approaches is to identify locations under the highest vulnerability or risk to one or multiple pressures. In this study, we adopted the conceptual approach of risk assessment by Holsman et al. (2017), complemented with numerical and spatial components from ecological and engineering frameworks, as we found them suitable considering the available datasets we had and that we further describe in this article (Cuevas et al., 2019; Eidsvig et al., 2011; Kaynia et al., 2008).

Despite previous studies about oil impacts on fisheries in sGoM, and some description of the conflicts between those industries, there is still a lack of spatially explicit, multi-source risk assessments integrating real oil detection data with spatial fishing effort indicators. This study aimed to quantify spatial risk and vulnerability of Fishing Fleets Space Use (FFSU) to oil-related pressures by integrating multi-source geospatial data and real oil detection records.

This is the first study that spatially assesses the vulnerability and risk of interactions between the FFSU and the oil industry in the sGoM. This assessment integrates local expert knowledge with systematic quantitative data to identify the zones of highest risk of interaction and the likelihood of conflicts. The information we produced is expected to help prioritize actions by decision-makers, according to their internal politics, protocols, and resources, to address the conflict between the oil and fishing industries (small-scale and industrial ones), to strengthen the institutional and civilian preparedness and response capabilities in case of an oil spill, as well as contribute to improving spatial information for decision-making regarding security surveillance for tackling increasing piracy, mostly to small-scale fisheries. It is also a foundation element necessary for local and regional maritime spatial planning to harmonize two different extractive activities that support the well-being of a considerable number of families in Mexico (Baltaoglu, 2025).

## Methods

**Fig. 1:**
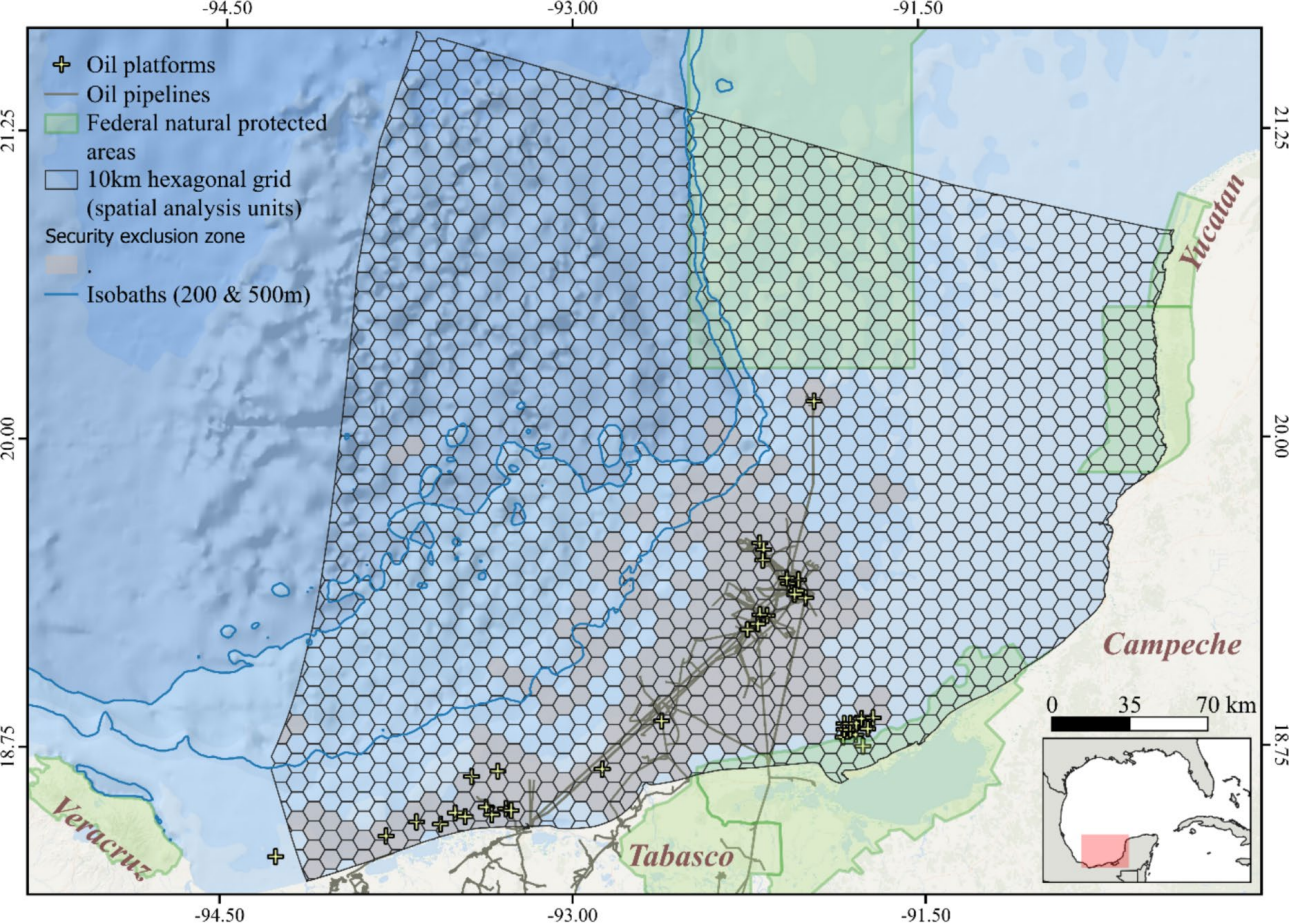
Geographical context of the maritime area where the oil and fishing industries coexist in the southern Gulf of Mexico

The study area was circumscribed by the shoreline of the Mexican states of Tabasco, Campeche, and part of the western coast of Yucatan to include the ports from where small-scale fisheries operate in the Sound of Campeche. The marine area extended offshore 200 km to include the region where most of the oil and gas marine infrastructure is located (Alpizar-Castro & Rodríguez-Monroy, 2016; Rodríguez-García et al., 2022), and it also included essential small-scale fishing grounds for fleets from the previously specified States and Tamaulipas (Crespo-Guerrero et al., 2019; Salazar-De-la-Cruz et al., 2020; Torres-Irineo et al.,  2021). The area was delimited by straight lines drawn from the shore at the state borders to the North and West (Fig. 1).

The analyses in this study were driven from a spatiotemporal perspective that incorporated data from multiple sources. All analyses described below were conducted using a combination of spatial analysis tools in open-source software, specifically QGIS, R, and Python. We used a hexagonal grid (vectorial file), each hexagon (spatial analysis unit) was 10 km in diameter, whose attribute table served to integrate and standardize all the variables for the analysis in a geographic information system (GIS) (Cuevas et al., 2019). We acknowledge that the size of the hexagons used for this first assessment of vulnerability and risk of FFSU to oil interactions in sGoM did not fit all the socioenvironmental processes of interest in the studied seascape, and in this article we present some of the existing interactions at the scale we defined, and when processes at different scales were detected, they should be addressed in further research. This approach has been well-documented as an effective and efficient strategy for integrating multiple data sources in a robust, versatile, and spatially explicit framework (Elayam et al., 2022; Rodríguez-Viñas et al., 2023).

### Vulnerability and risk assessments

In this study, you will find the results of implementing three different, but complementary, spatially explicit quantitative approaches. The first two approaches were described by Holsman et al. (2017) in their marine risk assessment approach, who described different classes and levels of analysis complexity depending on the data availability and their quality. We used approaches from their Level 2, which combines expert knowledge with geographic data for spatially assessing the potential interactions between FFSU and multiple threats associated with the oil industry. The first implemented approach (Class 1) intersects a single pressure (oil presence) and a single sensitive subject (FFSU), and the second approach (Class 3) analyzes multiple pressures (vessel transit, oil, and infrastructure presence) to a single sensitive subject (FFSU).

The third spatially quantitative approach assessed the risk of one sensitive subject (FFSU) to one pressure (oil on the sea surface), considering a pre-existing vulnerability. This approach is based on the general vulnerability equation described by Füssel & Klein (2006) and risk assessment described by Eidsvig et al. (2011).

We harvested data from distinct sources, including public databases, satellite imagery, and local expert knowledge mapping. All the distinct geographic layers were transferred to the hexagonal vectorial grid, and the algebraic operations for vulnerabilities and risk assessments occurred in their associated attribute table. The original data sources and satellite imagery used to build all the layers used in this analysis are public for replicating these analyses.

It is worth saying that the spatiotemporal distribution of fishing fleets is one of the significant historical gaps and a persistent challenge in fisheries science. In Mexico, the industrial fleet is obligated to use satellite monitoring systems, but the small-scale fisheries are not, so we took the best available geographic records to represent the spatial patterns of these fishing fleets; however, we acknowledge that this representation is imperfect, likely underestimated, and much more work is needed to increase and improve the availability of spatiotemporal data for both fishing fleets.

### Fishing seascape: the sensitive factor

We defined the areas occupied by small-scale and industrial fishing fleets (the FFSU) in our region of interest, by delimiting them using different data sources: local expert knowledge mapping, reported landings, publicly available data from satellite vessel monitoring systems (VMS), and the Global Fishing Watch database (Drakopulos et al., 2023). From now on, in this paper, we call FFSU those areas where we have participative or measured data that suggest an occupation of that area by a boat or vessel that is assumed to be engaged in some fishing activities. The three data sources were used to estimate an indicator of boat and vessel densities per hexagon, and the three sources were assembled to obtain the spatial configuration of the FFSU as the sensitive target variable. Each data source was individually rescaled from zero to one (0 to 1), so the three sources had the same quantitative value range and they were summed, then the output was usable for the rest of the vulnerability and risk assessments (Cuevas et al., 2019).

Lara-Mendoza et al. (2026) compiled 11,192 geographic locations of the fishing areas used by small-scale fishermen from Tabasco and Campeche, Mexico, during 9 years (2016–2024). They obtained the coordinates by direct collaboration with local fishermen and by interviewing the fishermen when they arrived at their port (participative mapping). Those assessed fleets reported to use gillnets, bottom longlines, trolling lines, and vertical longlines (for a description, read López-Rocha et al., 2021; Lara-Mendoza et al., 2026). The geographic data were incorporated into the hexagonal grid by estimating the density of records per hexagon.

The source of geographic data for delimiting the industrial FFSU was the publicly available satellite tracking data of the Vessel Monitoring Systems (VMS) reported to the Mexican fisheries authority in agreement with the Mexican Official Norm NOM-062-SAG/PESC-2014, which establishes the use of the satellite location and monitoring system for industrial fishing vessels in Mexico (Diario Oficial de la Federación, 2015). The fleets for which we compiled data in the study area held 794 industrial permits to capture finfish, tuna, shrimps, sharks, octopus, and swordfish, and the dataset covered the period 2017–2024, with a total of 437,631 geographic records for our study area. We used the *vms-analyzer* repository, which includes public libraries that are run in Python 3.12 (Python Software Foundation, 2024) (https://github.com/rcavieses/vms-analyzer), to standardize, filter, and analyze the VMS data. We standardized the recorded speed to kilometers per hour, and then we estimated the speed of the vessels to avoid errors in the original dataset. After eliminating stationary records of the vessels (0–1 km/h), mostly associated with their home ports, the rest of the navigation records were classified as fishing mode based on their speed (> 1 and < 4 km/h). We used as a reference the available studies on vessel satellite monitoring that defined industrial vessels’ speed when they were fishing, including a reference for the South Gulf of Mexico (Witt & Godley, 2007; Wakida-Kusunoki et al., 2010; Sameoto et al., 2025).

Finally, we did a query from the Global Fishing Watch (GFW) web portal mapper (Global Fishing Watch, 2026) to gather gridded counts of the number of detected boats assumed to be fishing based on their algorithms and quality controls for different satellite imagery (Kroodsma et al., 2018; Park et al., 2023). We downloaded a set of 1-km resolution rasters that contained the monthly frequency of boats estimated using night light detections (VIIRS satellite images) and radar vessel detections (SAR satellite images) for our study area from January 2017 to December 2023 (Hsu et al., 2019; Global Fishing Watch, 2026). Monthly rasters were summed to obtain an annual frequency layer and the abundance statistics at each hexagon.

GFW acknowledges that the vessel fishing activity they report in their products is designated as apparent, rather than certain, and accordingly, the information is provided “as is” without warranty of any kind (https://globalfishingwatch.org/terms-of-use/), and we adhere to this same disclaimer. GFW is often the best available, scientifically solid information about fishing effort in our oceans, and still, there remains a large knowledge gap about fishing efforts globally. We combined GFW data with other data sources of fishing efforts to delimitate the FFSU better, as those are the best available data sources for fishing effort in the region.

The three data sources (participative mapping, VMS and GFW data) were scaled from 0 to 1 in the same hexagonal grid, and then they were summed for each hexagon to get a cumulative value, which was rescaled from 0 to 1 again to obtain a final score of the FFSU. The hexagons with the highest values were assumed to have the strongest consensus score of representing FFSU sites.

### Approach no. 1: risk for one subject, one pressure approach (Class 1)

Following Holsman et al. (2017), we evaluated, in a one-to-one schema, the risk of interaction between the FFSU and the likelihood of the presence of oil on the sea surface. For this purpose, we used Eq. 1:

1 $$Risk=SensFFSU\times LkhdOil$$

where $SensFFSU$ is the composite layer of the sensitive factor FFSU, and $LkhdOil$ is the likelihood of satellite-detected oil on the sea surface. This equation was applied to each hexagon.

The likelihood of detecting oil on the sea surface (0 to 1) was estimated by dividing the number of satellite oil detections (2018–2024) by the number of satellite images analyzed in the same period, at each hexagon (Eq. 2):

2 $$LkhdOil=N\;Oil\;Detections/N\;Satellite\;Observations$$

where, for each hexagon, $N\;Oil\;Detections$ is the number of detected oil polygons in the period 2018–2024, and $N\;Satellite\;Observations$ is the number of available satellite images that were processed, that is, the number of satellite surveys at each hexagon.

Then, we multiplied the layer of the FFSU (sensitive factor) by the layer of the likelihood of detecting oil to obtain a direct risk estimation of the interaction between FFSU and oil on the sea surface.

The context for the identification of oil spills in our study area was derived from the operational oil spill monitoring program for sGOM described by Uribe-Martínez et al. (2024). Briefly, the system includes the interpretation, by expert personnel, of public satellite imagery to identify backscatter anomalies associated with oil on the sea surface (Sun et al., 2015; Uribe-Martínez et al., 2024). This technique allows for highlighting and delimiting the detected spectral anomalies associated with the presumed oil on the sea surface, including a variety of backscatter responses going from oily waters to thicker layers of oil on the surface. The oil on the sea surface reflects very differently depending on the atmospheric and oceanic conditions, which is why expert supervision is used and helps to detect anomalies even under unfavorable conditions such as sun glint, fog, or clouds (Uribe-Martínez et al., 2024).

We built a geographic layer with values of the likelihood of oil presence on the sea surface, from a total of 1631 analyzed satellite images from 2018 to 2024, acquired by the sensors Sentinel 1-SAR, Sentinel 2-MSI (https://cophub.copernicus.eu/), as well as Landsat 8 and 9 OLI (https://earthexplorer.usgs.gov/), and delimited polygons of oil spills (Uribe-Martínez et al., 2024).

### Approach no. 2: cumulative vulnerability for one subject to multiple pressures (modified from Class 3)

It is acknowledged that any natural or anthropic system is mostly under multiple concurrent sources of pressure that configure its vulnerability to other potential threats, such as oil spills. The more system elements are included in the vulnerability and risk assessments, the larger and finer the resolutions of the datasets must be obtained to fulfill all the components of the equations (Holsman et al., 2017).

In this study, at each hexagon, we evaluated the cumulative vulnerability of the composite FFSU to multiple pressures (*pr*) associated with the oil industry. We used (Eq. 3) to calculate the cumulative vulnerability (modified from Füssel & Klein 2006 by Cuevas et al., 2019):

3 $$CumVulnFFSU=\sum\nolimits_{pr=1}^n\;SensFFSU\times{Exp}_{pr}-{SC}_{pr}$$

where *CumVulnFFSU* is the cumulative vulnerability of the FFSU to interact with the *n* pressures (pr); *SensFFSU* is the sensitive factor represented by the composite FFSU, *Exp*_*pr*_ is the exposure to the *nth* pressure, and *SC*_*pr*_ is the stability coefficient of the FFSU to the pressure *pr*.

We assessed four pressures associated with oil industry activities that are likely to interact with the composite FFSU directly or indirectly: the presence of oil platforms, wells, transit of large vessels, and pipelines. The location and density of platforms, wells, and submarine pipelines represented a potential source of negative interactions because (1) they are barriers to maritime transit or fishing activities since the Mexican law banned any activity or maritime transit not related to the oil industry in a buffer of 500 m around those facilities (DOF, 2019); and (2) because several of them have been reported to be the source of oil spills in that region of interest (Uribe-Martínez et al., 2024).

In this study, the vulnerability of small-scale FFSU to the presence intensity of industrial vessels was included because of the potential direct interaction between a ship and a fishing boat or its fishing gear. Also, the vessels are associated with the presence of oil on the sea surface because there are records of the spills related to industrial vessels in this region. We acquired the vessel’s transit data from the Nationwide Automatic Identification System (AIS) 2021 (Office for Coastal Management, 2024) for the sGoM as a sample of the spatial general pattern of vessel transit routes in our study area. The vessels’ tracks are gathered by their AIS and filtered to a one-minute frequency rate, with a subset to depict the location and description of vessels located within the study area. The AIS vessels’ data were downloaded as a raster image containing the number of trajectories recorded in each pixel; then, we used QGIS (QGIS.org, 2025) to calculate the frequency of trajectories per hexagon.

Also, we obtained the 2023 official public layers of oil platforms, wells (active and inactive), and pipelines from the webpage of the closed National Commission of Hydrocarbons in Mexico (DOF, 2025). Some additional locations of oil platforms were obtained from previous oil spill monitoring efforts in our study area (Uribe-Martínez et al., 2020). We quantified the abundance of wells and platforms in each hexagon. All the abundance values of the above-described pressures in the hexagonal grid were rescaled from 0 to 1 so that they would be in the same order of magnitude as the fishing grounds layer.

Regarding the stability coefficient (Cuevas et al., 2019), the security zone around the oil industry facilities intends to give security to the oil industry and any boat close to the areas where the associated cargo vessels maneuver (Quist & Nygren, 2015); nevertheless, they also represent a driver of the conflict between the two industries, and it is assumed to show the asymmetry in alliances and dominance (Aarflot et al., 2024). Globally, since the oil industry was established offshore, it was clear that, except for the control of pollution, the law is an instrument incapable of meeting the needs of the two sectors (Grant, 1978) and ignored the fishers’ security.

We acknowledge the controversy of the effectiveness of such security zones to reduce risks to the fishing fleets; nevertheless, in this study we used them as a factor that was considered a positive component as the stability coefficient in the vulnerability equation (Eq. 1). We created the polygons of the government decree for the security zones (DOF, 2019) using a buffer of 500 m around any oil industry facility, as it is defined by law, and intersected them with the hexagonal grid. We assigned a 25% vulnerability reduction in those hexagons that intersected the security zones (Cuevas et al., 2019; Liceaga Correa et al., 2021).

Finally, we solved Eq. 3 for each of the four previously described pressures (platforms, pipelines, vessels, and wells) as sources of potential interactions with the FFSU. For each pressure, we used the same sensitivity layer of FFSU, multiplied by the values of the individual one, minus the stability coefficient represented by the security zones. In this study, we reported the specific vulnerability of FFSU for each of the analyzed sources of potential interactions.

Then, after we obtained the individual vulnerability of FFSU to each of the four pressures associated with the oil industry that we evaluated, based on statistics from the monitoring system of oil presence on the sea surface (Uribe-Martínez et al., 2020; 2024), we estimated a weighting factor for each of the pressures. We used the Analytic Hierarchy Process (AHP) by Saaty (2008), which assesses hierarchy criteria and alternatives, to calculate the weight of each pressure by pairwise comparisons of the four potential sources of oil on the sea surface, based on the frequency of reported oil spills associated with each pressure. We obtained their relative weight as sources of oil: platforms (*w* = 0.521739), wells (*w* = 0.260869), pipelines (*w* = 0.130434), and vessels (*w *= 0.065217). The vulnerabilities of the FFSU to each of the individual pressures were multiplied by their corresponding constant weighting value; then, the four specific weighted vulnerabilities were algebraically summed to get a hexagonal grid of the cumulative vulnerability of the fishing grounds (Eq. 3).

### Approach no. 3: risk for one subject, one threat, considering pre-existing vulnerability—case study

For assessing the risk of the FFSU interacting with oil, under a pre-existing cumulative vulnerability condition, we utilized the Eidsvig et al. (2011) approach. We considered the cumulative vulnerability of the FFSU, the likelihood of the threat occurring (the oil on the surface in this case), and the estimated losses when the interaction between oil and fishing grounds occurs.

We could only acquire the information needed to implement this approach for the industrial fishing fleet (VMS datasets). We evaluated the vulnerability of FFSU using Eq. 3 (modified from Füssel & Klein 2006 by Cuevas et al., 2019). Then, we multiplied the estimated cumulative vulnerability by the likelihood of oil presence and by a cost score of the interaction of oil with fishing activities (Eidsvig et al., 2011),

4 $$Risk =CumVulnFFSU\times LkhdOil\times Loss$$

where $Risk$ is defined as the measure of the risk of negative interactions between the FFSU industrial fleets and oil presence, and *Loss* is an estimation of the presumed economic losses on the FFSU in case of oil impacting those areas (modified from Eidsvig et al., 2011). The cumulative vulnerability for the industrial FFSU was calculated as described in Eq. 3, and the likelihood of oil presence was the same layer created with Eq. 2.

The spatial distribution of economic value across the industrial FFSU was quantified using a two-stage methodological framework to assess the potential costs of interactions with oil spills. The monetary value per operational hour $Vh$ was calculated with the following equation:

5 $$Vh=VTot/HTot$$

where $VTot$ is the summed fisheries value estimated from the publicly available fishing arrival reports (2021–2023) (https://conapesca.gob.mx/wb/cona/avisos_arribo_cosecha_produccion), and $HTot$ is the total fishing time in hours per unit area for each hexagon. The time was calculated by the difference in time between the maximum and minimum timestamps (in hours) of the analyzed VMS records in each hexagon. The economic value for each hexagonal spatial unit ($EV$) was then computed as follows:

6 $$\mathrm{E}\mathrm{V}=Vh\times T$$

where $\mathrm{T}$ is the sum of time spent per vessel in each hexagon, and it was estimated from the VMS data using speed filtering criteria of 0–4 km to isolate active fishing operations.

The resulting values were normalized and expressed in millions of USD (considering the 2023 prices per kilogram for each caught species) and then rescaled to values from 0 to 1 to enable comparative analysis of economic importance across fishing grounds. This methodology assumes a linear relationship between vessel operation time and profit within each spatial unit, facilitating the spatially explicit allocation of monetary value based on the distribution of fishing effort.

### Geographic approaches assemble

To contribute an integral map of the highest vulnerable and at-risk areas, the hexagons with values higher than 0.75, the fourth quartile of the data distribution, for each of the three quantitatively evaluated approaches were labeled as 1. Finally, we obtained the frequency of each hexagon having the highest vulnerability and risk values. This output shows the consensus seascape of vulnerability and risk for the FFSU to oil industry activities.

## Results

### Composite sensitive fishing seascape

Those areas where the highest FFSU from the different data sources occurred had the highest sensitivity value (1 or the closest), because it was assumed that those areas would host the highest fishing use; and on the other hand, the lowest FFSU occurrence data in each location, the lowest sensitivity, and the least expected fishing use.

**Fig. 2:**
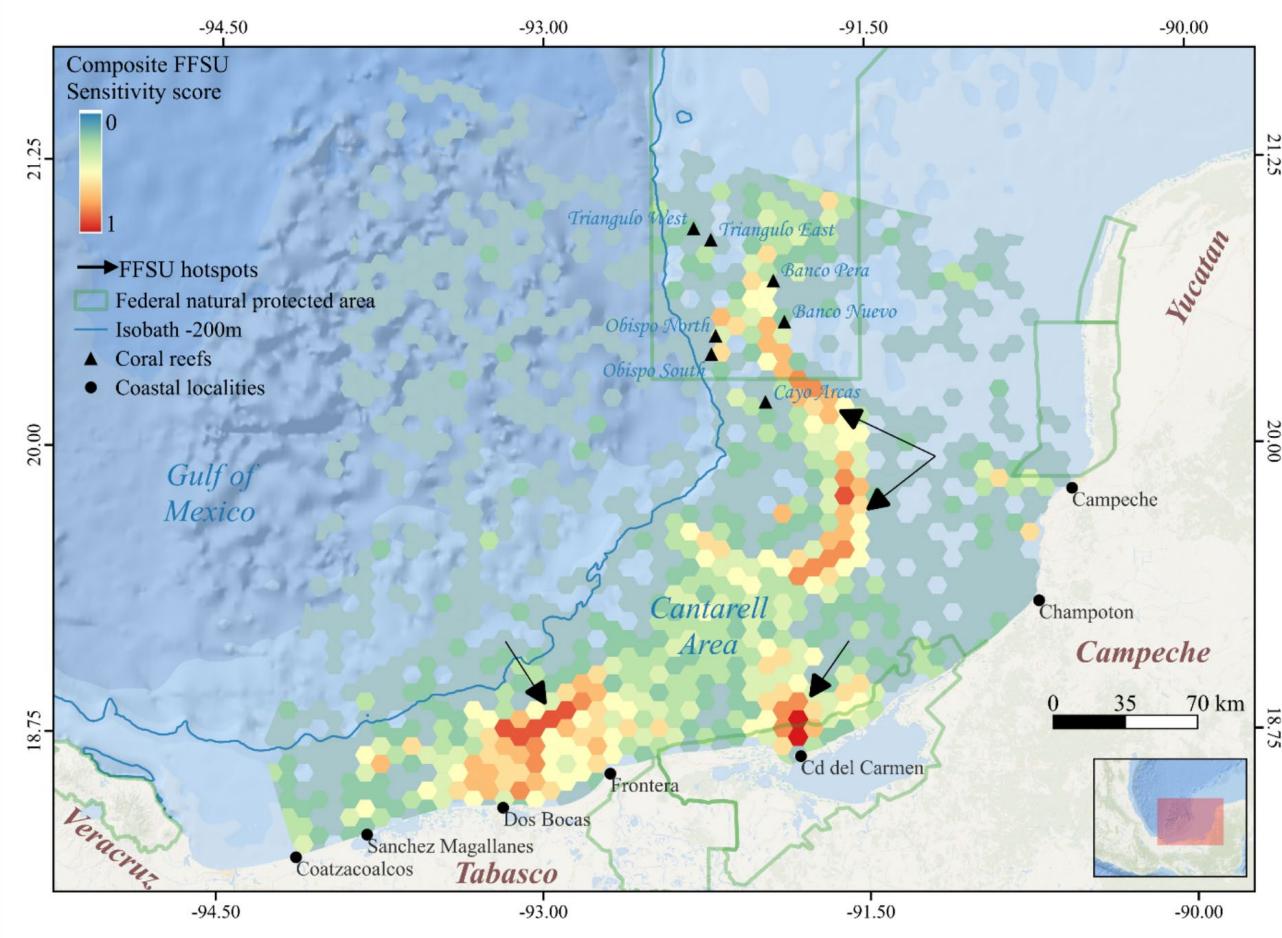
Fishing Fleets Space Use (FFSU) sensitivity derived from participative mapping, satellite detections (Global Fishing Watch), and on-site location inference for small-scale and some industrial fleets from coastal localities in the southern Gulf of Mexico. The black arrows sign some FFSU hotspots

We identified three highly sensitive hotspots, one in front of Campeche at approximately 100 km offshore (Fig. 2), the second in front of Cd. del Carmen, where significant small-scale fishing efforts take place, and the last one in front of Tabasco, between the localities of Frontera and the west of Dos Bocas Port. The decreed restriction area for fishing around the oil industry facilities showed low sensitivity, as the fishing industry does not systematically use them. The presence of boats in that area is addressed in the “Discussion” section.

The FFSU hotspot in front of Campeche’s central littoral (northernmost arrows in Fig. 2) is part of an FFSU corridor that reaches the federal natural protected area named Reefs of the Southern Gulf of Mexico (recently decreed), where essential rocky reefs are located (Obispo and Triangulo Cays). In Cayo Arcas is located the most significant marine sales point for Pemex, and it is just south of the latter natural protected area (Fig. 2) but still immersed in the main small-scale and industrial fishing seascapes.

### Approach no. 1: risk for one subject, one pressure approach

**Fig. 3:**
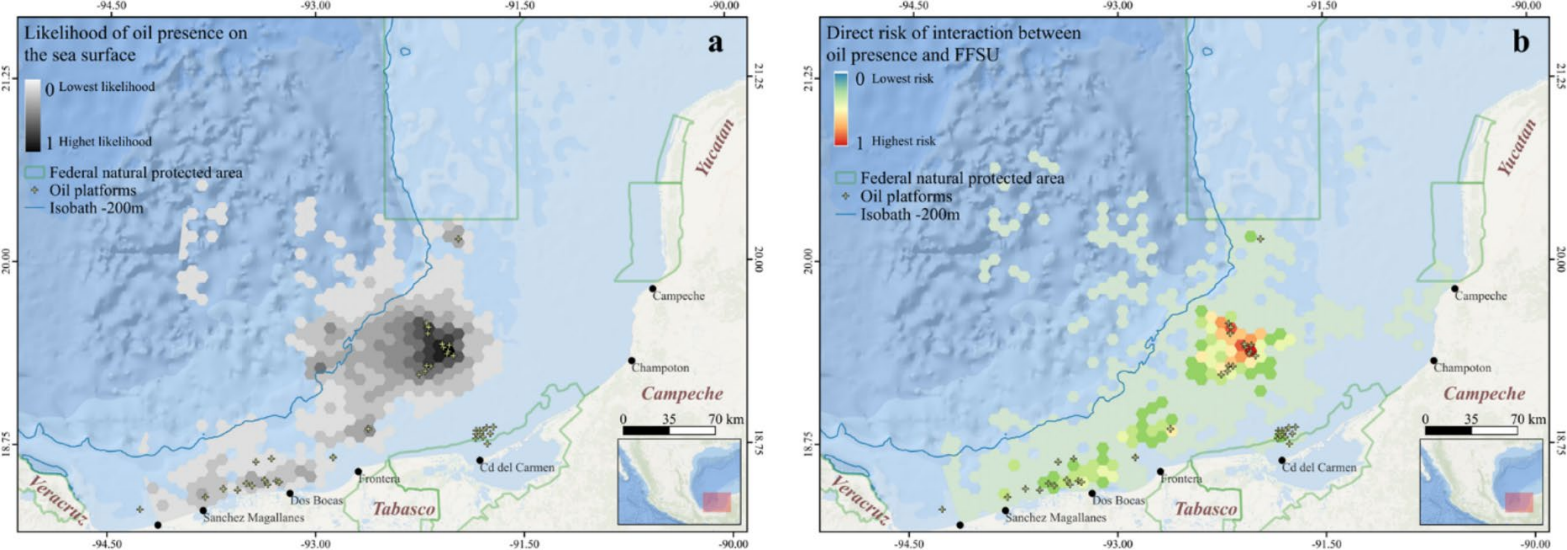
The risk elements of Eq. 1 to evaluate the potential interaction between the likelihood of oil presence on the sea surface (**a**) and the FFSU used by industrial and small-scale fleets in the sGoM to obtain a risk score (**b**) in the study area

The satellite-detected presence of oil covered a wide zone in the study area, with the highest likelihood associated with the Cantarell zone, where most of the platforms and ducts are located (Fig. 3a). Those areas close to a value of zero (0) had the lowest likelihood of encountering oil anomalies on the sea surface when a satellite image of the study area is analyzed. Those areas in black (value 1) had the highest likelihood of finding a spectral anomaly associated with oil on the sea surface when analyzing a satellite image.

Tabasco’s coast is highly likely to have oil presence, and together with Cantarell, they had the highest risk of interactions with FFSU (Fig. 3b). Those hexagons with the highest values (close to 1) of risk of FFSU and oil interacting are the areas where a combination of relatively high likelihood of oil presence and FFSU occurred, so those are the areas of most significant concern for marine spatial planning, and it is also an indicator that informs decision-makers the territories where social conflicts are more likely to emerge.

In Tabasco’s waters, the spatial configuration of the oil industry infrastructure and its security zones represent a geographic barrier for Tabasco’s fisheries (Fig. 3).

### Approach no. 2: cumulative vulnerability of FFSU

**Fig. 4:**
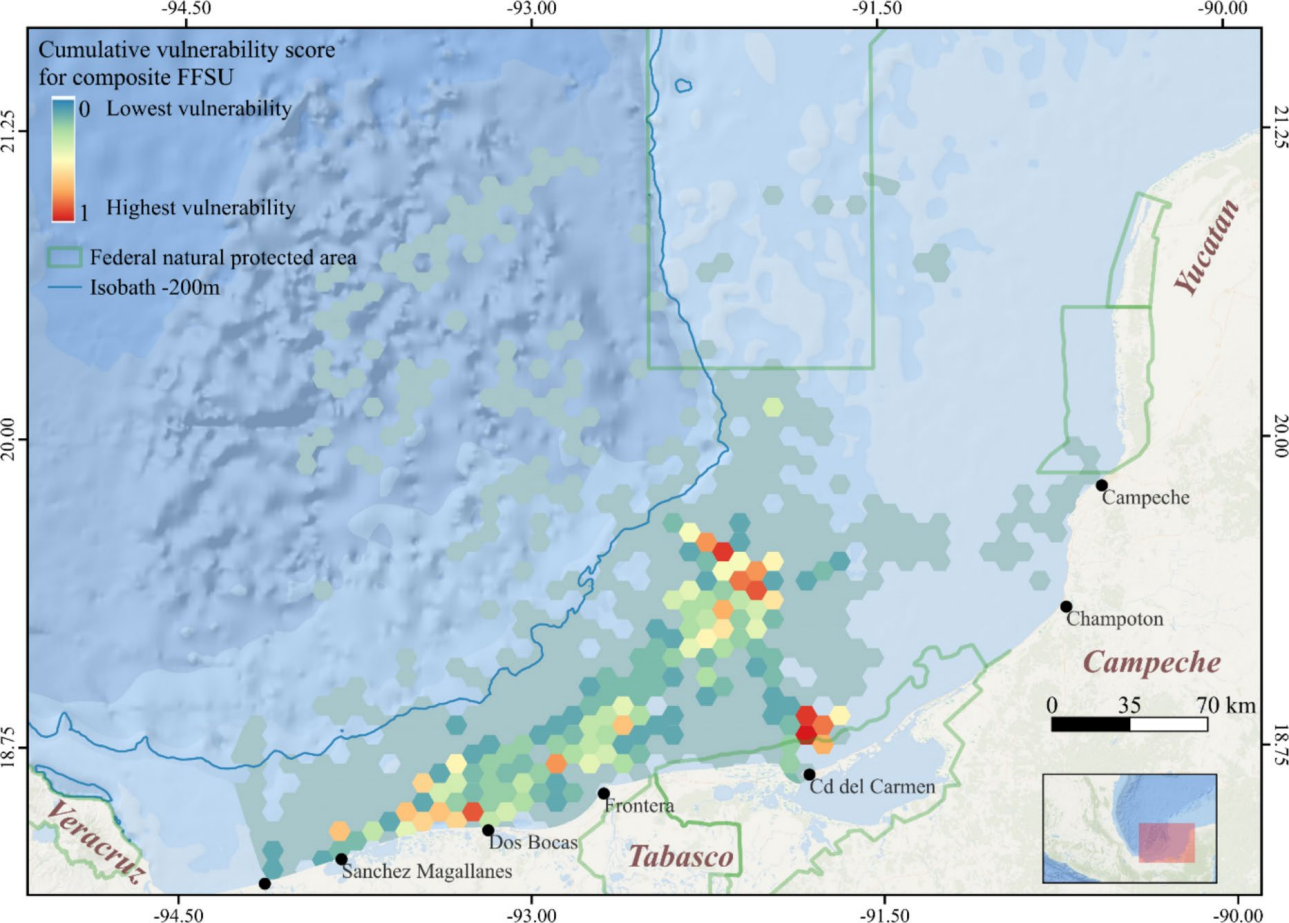
Spatial configuration of the cumulative vulnerability of FFSU for small-scale and industrial fleets in sGoM to potential interactions with the oil industry infrastructure and the presence of oil on the sea surface

We obtained individual vulnerability estimates for each pressure (Fig. S1) and an indicator of cumulative vulnerability for all of them (Fig. 4). Its spatial configuration showed the Cantarell zone and the marine zone in front of Tabasco as the ones with the highest vulnerability to the oil industry infrastructure and the presence of oil.

Except for the Cantarell zone, the security area for the oil industry had a low vulnerability score, as the fishing industry only occupied those areas under exceptional circumstances, some of which are further discussed in this article. The zone of highest vulnerability close to shore between Dos Bocas and Sanchez Magallanes configured a seascape that restricts the mobility of the small-scale fleets from that shoreline segment (Fig. 4).

### Approach no. 3: risk for industrial fishing grounds—case study

**Fig. 5:**
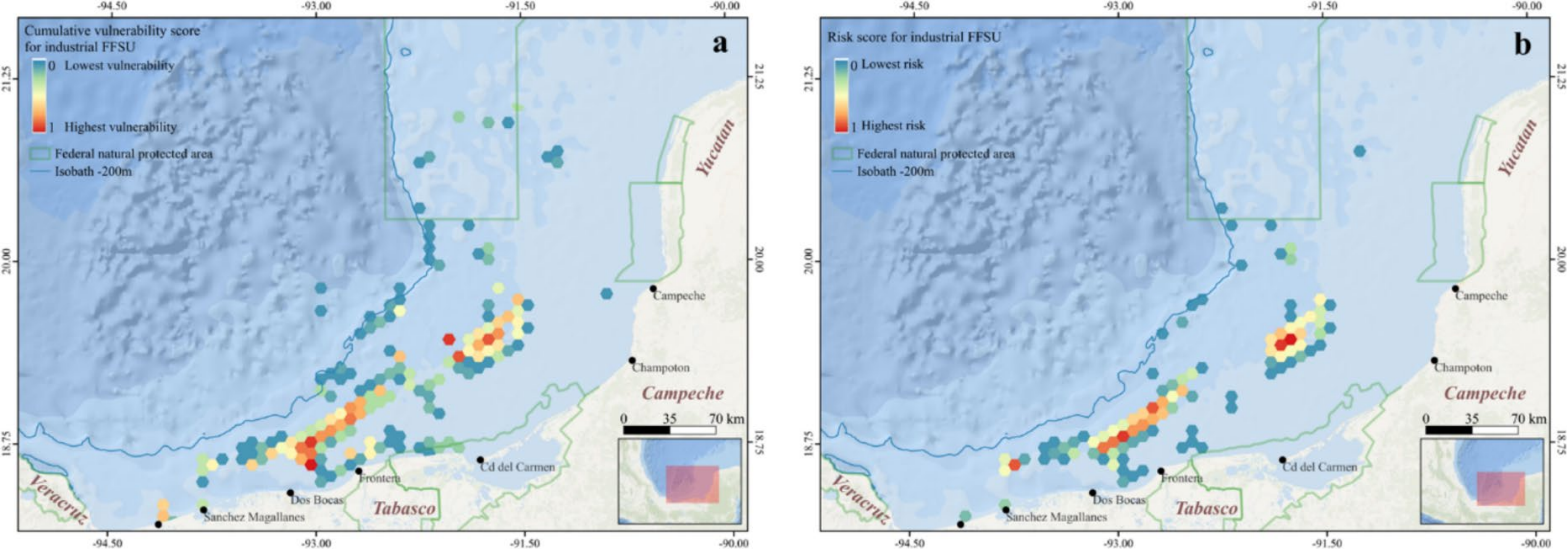
Case study of the industrial FFSU’s cumulative vulnerability (**a**) and the output risk score to the presence of oil on the sea surface (**b**) in the sGoM

Two zones with the highest risk for industrial FFSU were identified, one in front of the coast of Tabasco and another to the north of Cd. del Carmen (Fig. 5b).

Once again, the oil industry infrastructure and the cumulative vulnerability and risk values for the FFSU in front of Tabasco configure a seascape that represents a barrier for the local fishing activities.

### Geographic approaches assemble

**Fig. 6:**
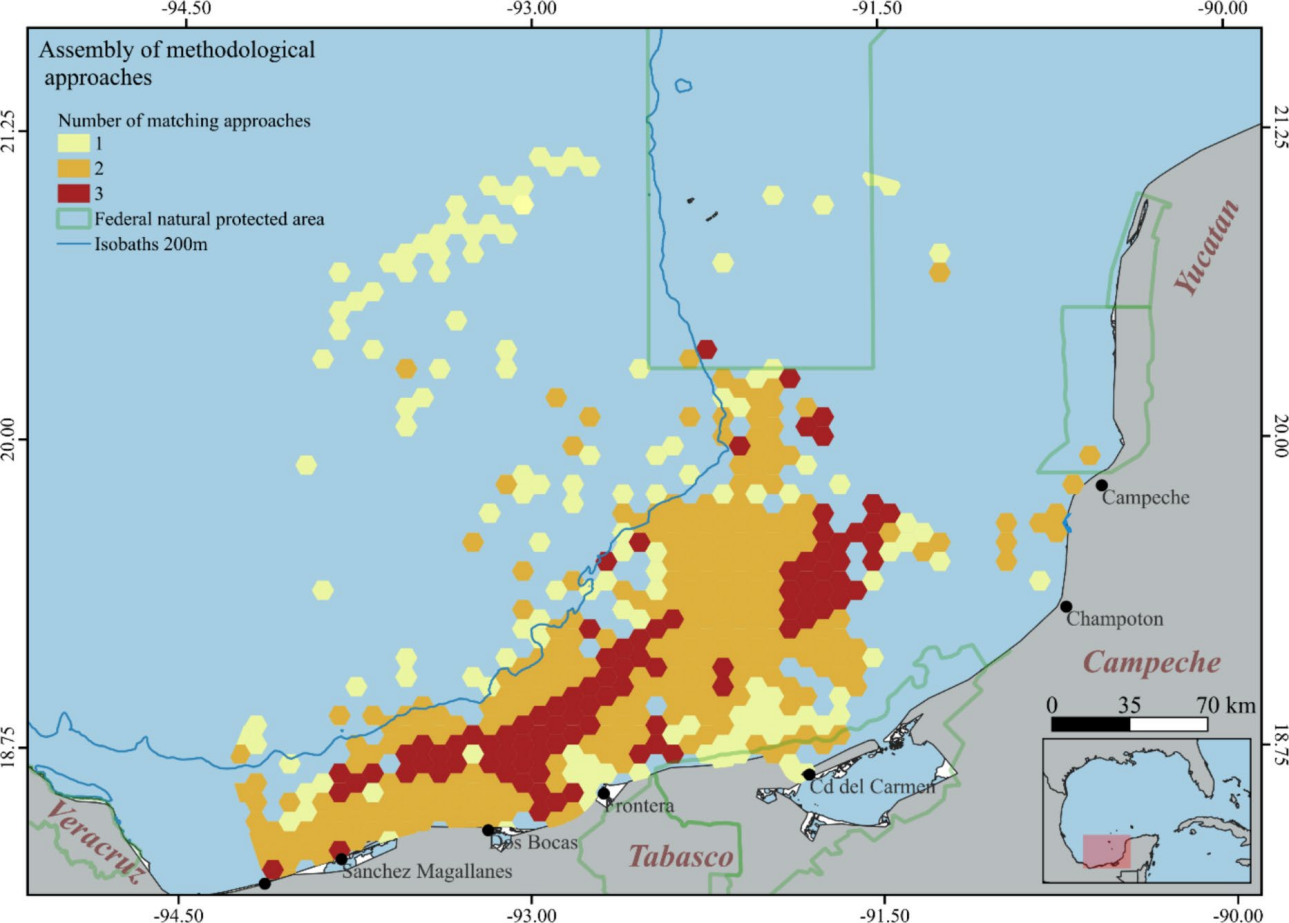
Spatial configuration of the risk and cumulative vulnerability consensus for the FFSU to interact with oil industry pressures and threats

Finally, from Sanchez Magallanes to Champoton, the continental shelf was extensively covered by zones where potential interactions between the oil industry facilities (wells, platforms, pipelines, or vessels), or the presence of oil, may interact with an FFSU (Fig. 6). Two large zones are discernible: the Cantarell complex and the marine zone in front of the Tabasco coast (in dark red, Fig. 6), where the three vulnerability and risk estimations coincide. The latter is of the greatest concern because its configuration runs parallel to the coastline and represents a potential mobility barrier for fishing fleets in the region. Although it is discussed later, the central region, where the security zones are located, represents areas where the FFSU has lower vulnerability and risk interacting with the oil industry because the security zones themselves have blocked and displaced fishing fleets in those regions.

## Discussion

The diversity of available datasets has become an opportunity for assessing the vulnerability and risk of potential interactions between the fishing and oil industries from different perspectives. Here, we presented three quantitative complementary approaches for generating information to strengthen the decision-making and spatial planning processes. This methodological blend improves the available knowledge and decision tools for disaster risk management, highlighting its simpleness, versatility, and reliability in identifying the risk that occurs in complex contexts (Eidsvig et al., 2011; Gibbs & Browman, 2015; Stelzenmüller et al., 2015; Holsman et al., 2017).

These first-order assessment approaches are particularly crucial for emergent economies with limited financial resources and a tendency to prioritize investment in response and recovery over prevention. In Latin America and the Caribbean alone, disasters are increasingly frequent and devastating, as the region accounts for 53% of the world’s economic losses due to disasters (United Nations Office for Disaster Risk Reduction, 2025). In this context, the critical need for planning and monitoring accessible tools to mitigate and respond to adverse situations cannot be overstated.

Geospatial technologies and techniques (geographic information systems, remote sensing analyses) have become essential for oil spill preparedness and response, strengthening the definition of strategies and their implementation for in-charge command authorities (Temitope-Yekeen & Balogun, 2020). Together with Uribe-Martínez et al. (2024), the spatially explicit information contributed by this article will aid local and regional response strategies that are defined in the Mexican national response plan to oil spills (Diario Oficial de la Federación, 2023), as it can also serve non-governmental operational systems, like the International Charter Space & Major Disasters (https://disasterscharter.org/), that give the decision-makers scientific information for better-informed management actions. Such independent efforts are critical in countries with incipient transparency regarding their oil industry regulations (Li et al., 2025). For instance, in 2023, in Mexico, Pemex suffered the loss of 1,371 barrels of oil from the Balam-TA platform (PEMEX, 2023).

The more outputs, like those we contributed to in this research, that are used for decision-making and marine spatial planning strategies, the better opportunities for setting stronger negotiations about the shared seascape, aiming at the highest common benefit for the long-term viability of the socioecosystem. Any negotiating strategy needs basic spatially explicit information for starting the dialogue among the parties, and that is what this research contributed to, for a better coexistence in highly productive seascapes such as the sGoM.

### Vulnerability and risk of FFSU

When assessing vulnerability or risk to single or multiple pressures, it is highly desirable to include the evaluation of the pre-existing condition of the system of interest (Tyack et al., 2022). That was the foundation for us to assess the specific vulnerability of interactions between the FFSU and the oil industry elements (platforms, ducts, wells, vessels), and then evaluate the cumulative vulnerability to the presence of oil.

We found the highest vulnerability and risk values mostly outside the security zones imposed around the oil industry infrastructure, mainly because there is no vulnerability or risk without the exposure of the sensitive element. Inside the security zones, the presence of fishing boats is fortuitous, unexpected, and can reflect the fishermen’s need to access refuge and resting zones when they are so far from land. In some cases (Figs. 3 and 4), some high-risk and cumulative values for FFSU are presented close to oil industry facilities, and that is mainly due to the high likelihood of oil presence in those zones. No matter if the low fishing occupancy values were there, some of those zones stood out as the highest risk and vulnerable zones because of high probability of oil presence. The areas where the security zones are located have a medium or low consensus score (Fig. 6) because they are not primarily used for fishing activities. In terms of marine management, those high-risk and vulnerability areas inside security zones should not be an issue of concern, given the decreed laws. Nevertheless, this study highlights evidence of some small-scale and industrial space occupancy that should be investigated in greater detail and with distinct approaches than those implemented in this study.

We remark that such an asymmetric and prohibitive strategy opposes a transparent, inclusive, and human management tool. As previously stated, the security zones are primarily intended to protect the oil industry infrastructure from piracy and terrorism, to protect submarine infrastructure that could be damaged by fishing gear, and to protect human life in the sea (Diario Oficial de la Federación, 2019). Nevertheless, their expansion has fomented historic socioenvironmental conflicts in the region, along with a historical lack of intersectoral dialogue for co-constructing a balanced, harmonized seascape management.

Security exclusion zones have been discussed mainly from the perspective of international law and human rights (Zalik, 2009; Kashubsky & Morrison, 2013). It has been claimed that there is a need for other security models that can be efficient and protect the fishing vessels from piracy in the region where both industries coexist (Ramos-Muñoz et al., 2019). The dialogue for participative ordering of the marine territory has been significantly enhanced by tools such as agent-based models. These tools promote gamification strategies that have proved to be efficient, inclusive, and iterative for reaching consensual agreements between stakeholders with sole objectives (Ge et al., 2018; Keijser et al., 2018; Müller et al., 2013). Such strategies represent opportunities for opening and strengthening the multisectoral participative dialogue in this region of coexistence between the fishing and oil industries.

Regarding the novelty of the results presented in this vulnerability and risk study, we used actual satellite detections of oil, including those associated with recent verified oil spills, instead of oil dispersion models (Bayramov et al., 2018; Pavlov et al., 2022). This feature is essential because the certainty for the areas where the oil was detected is higher than in the output of a numerical model, and it also supports its robustness and relevance for preparedness and response in the face of an oil spill contingency.

Disasters particularly impact vulnerable and marginalized populations, with a significant severity on the poorest and most excluded individuals (United Nations Office for Disaster Risk Reduction, 2025). In the case of sGoM, during the last five decades, the marginalized fishing communities have struggled with the creation of exclusion fishing zones along with new oil and gas extraction infrastructure (Montejo-Damián et al., 2022). In this regard, a critical knowledge gap remains: a holistic evaluation of the cumulative vulnerability of humans’ livelihoods and their wellbeing, linked with the essential ecosystems in the sGoM. Such an analysis will boost the decision-making capabilities for more comprehensive management policies as part of Mexico’s preparedness and response protocols (Holsman et al., 2017). Nevertheless, the present study stands as the first contribution assessing vulnerability and risk for a key economic activity and opens the opportunity to integrate the analyses with previous ecological vulnerability and risk assessments produced for the sGoM (Cuevas et al., 2019; Saldaña-Ruíz et al., 2020; Sosa-Nishizaki et al., 2020).

There is a relevant need for respecting the right of secure working conditions for small-scale fishermen in sGoM, a condition that is exacerbated by the fact that they must travel hundreds of kilometers to reach their fishing grounds. In the case of small-scale fisheries, we strongly suggest public and private co-investments to implement satellite technology that gives them and their families more security and dignity in their jobs. Some examples exist for the sGoM that could be replicated and expanded in the broader region (Torres-Irineo et al., 2021; Uribe-Sandoval, 2025). This technology will also contribute to operational geographic data, together with logbook records of their catches, so that a formal standardized risk assessment can be done for that industry (Gibbs & Browman, 2015). In the sGoM, there are previous efforts to delimit the fishing grounds used by small-scale fleets (Quijano-Quiñones et al., 2021; Torres-Irineo et al., 2021), but there are still several key fleets in that same region that must be characterized. The implementation of such satellite communication from small-scale fleets will also open an opportunity for advancing maritime security and metocean operational monitoring by joining the volunteer observing ship program (VOS) promoted by the World Meteorological Organization, which has contributed large datasets for around the world (Jiang et al., 2019).

This study has three main limitations. First, the spatial resolution defined by the hexagonal units may obscure fine-scale interactions, such as vessel proximity to platforms and the duration of these encounters. Second, the input datasets have inherent limitations related to their origin (e.g., GFW disclaimers), which introduce uncertainty into the results despite representing the best available information. Third, the vulnerability and risk mosaics represent only instantaneous snapshots of conditions in the study area. Repeated, systematic assessments are therefore needed to support operational risk management, particularly given the increasing frequency of oil spill events in the region.

### Increasing resilience: contributions to harmonizing the complex coexistence

The primary outcome of this spatial information directly contributes to updating the territory planning for the sGoM (Diario Oficial de la Federación, 2012). It is also expected that these results will lead to strategies for achieving agreements on how the restriction zones can allow transit for small-scale fleets. Currently, fisheries are being enclosed by the growing oil industry infrastructure, forcing them to travel longer distances to reach distant remnant fishing grounds, increasing their vulnerability and risk (Ramos-Muñoz et al., 2019).

We contributed with some essential geographic layers needed to achieve consensus on territory planning with approaches, such as agent-based analysis, spatial marine planning (Ge et al., 2018; Lindkvist et al., 2020). It is also important to note that our collaboration with local communities achieved with this assessment addresses the relational dimension with academia, and we look forward to increasing the institutional capabilities for addressing this conflictive coexistence.

Regarding conflict solutions, Kusters et al. (2025) described the ‘institutional capacities’ as a key factor for conflict resolution. Together with Trygg and Wenander (2022), they described four dimensions of the IC: knowledge resources (relevant data and information needed for the planning contexts), relational resources (stakeholders and their interactions, networks), mobilization capacity (capabilities of actors to use the knowledge and networks for implementing resolution actions), and institutional space (the institutional freedom for adopting new approaches for solving conflicts). With this study, we contribute to at least the first dimension.

Since the onset of offshore oil spills, the central challenge has been accessing relevant information and effective monitoring. As demonstrated here, technological advancements have made it possible to integrate data. However, it is essential to strengthen alliances with legislative systems to equilibrate the state-oil/fishing industries alliances that have been asymmetrically eroded over nearly a century.

## Conclusion

In the context of the high interest in marine spatial management and governance, particularly where historical conflicts occur, the information we contribute from this research serves decision-makers for strengthening their institutional preparedness, response, and remediation actions in case of oil industry incidents. Identifying and describing Fishing Fleets Space Use (FFSU) with the highest risk of interacting with oil on the sea surface is a critical step to minimizing disasters and buttressing the resilience and sustainability of sGoM. Notably, the obtained outcomes contribute to spatial knowledge about the territorial conflict between the oil and fishing industries.

This regional assessment describes the seascape used by coastal communities with distinct socioeconomic conditions, contributing to leading the efforts for strengthening their preparedness, response, and management protocols in case of oil spills, or any other incident associated with the oil industry.

The results and discussion in this study contribute to an assemblage of scientific and technical information that jointly serves as essential social (Oliveto-Andrade et al., 2024), operational (Uribe-Martínez et al., 2024), governance (Salazar-De-la-Cruz et al., 2020), and spatial marine management (this study) for a region where historical key extractive industries for Mexico coexist.

## Supplementary Information

Below is the link to the electronic supplementary material.

ESM 1 (556 KB DOCX)

## Data Availability

Data sets generated during the current study are available from the corresponding author on reasonable request.

## References

[CR1] Aarflot, J. M., Bjørdal, V. R., Dunlop, K. M., Espinasse, M., Husson, B., Lindstrøm, U., Keulder-Stenevik, F., Ono, K., Siwertsson, A., & Skern-Mauritzen, M. (2024). Ecosystem risk from human use of ocean space and resources: A case study from the Norwegian coast. *Ocean and Coastal Management,**256*, Article 107299. 10.1016/j.ocecoaman.2024.107299

[CR2] Alpizar-Castro, I., & Rodríguez-Monroy, C. (2016). Review of Mexico’s energy reform in 2013: Background, analysis of the reform and reactions. *Renewable & Sustainable Energy Reviews,**58*, 725–736. 10.1016/j.rser.2015.12.291

[CR3] Andrews, N., Bennett, N. J., Le Billon, P., Green, S. J., Cisneros-Montemayor, A. M., Amongin, S., Gray, N. J., & Sumaila, U. R. (2021). Oil, fisheries and coastal communities: A review of impacts on the environment, livelihoods, space and governance. *Energy Research & Social Science,**75*, Article 102009. 10.1016/j.erss.2021.102009

[CR4] Baltaoglu, S. (2025). Prospects of marine spatial planning in Türkiye. *Regional Studies in Marine Science,* Article 103997. 10.1016/j.rsma.2024.103997

[CR5] Bayramov, E., Kada, M., & Buchroithner, M. (2018). Monitoring oil spill hotspots, contamination probability modelling and assessment of coastal impacts in the Caspian Sea using SENTINEL-1, LANDSAT-8, RADARSAT, ENVISAT and ERS satellite sensors. *Journal of Operational Oceanography,**11*(1), 27–43. 10.1080/1755876X.2018.1438343

[CR6] Bethel, M. B., Braud, D. H., Lambeth, T., Dardar, D. S., & Ferguson-Bohne, P. (2022). Mapping risk factors to climate change impacts using traditional ecological knowledge to support adaptation planning with a Native American tribe in Louisiana. *Journal of Environmental Management,**301*, Article 113801. 10.1016/i.envman.2021.113801 34600422 10.1016/j.jenvman.2021.113801

[CR7] Bethel, M. B., Brien, L. F., Esposito, M. M., Miller, C. T., Buras, H. S., Laska, S. B., Philippe, R., Peterson, K. J., & Richards, C. P. (2014). Sci-TEK: A GIS-based multidisciplinary method for incorporating traditional ecological knowledge into Louisiana’s coastal restoration decision-making processes. *Journal of Coastal Research,**30*(5), 1081–1089. 10.2112/JCOASTRES-D-13-00214.1

[CR8] Camara, F. S., Pinto, F. R., da Silva, F. R., Soares, M., & De Paula, T. M. (2021). Socioeconomic vulnerability of communities on the Brazilian coast to the largest oil spill (2019-2020) in tropical oceans. *Ocean and Coastal Management,* Article 105506. 10.1016/j.ocecoaman.2020.105506

[CR9] Chisadza, C., Clance, M., Gupta, R., & Wohar, M. E. (2024). Giant oil discoveries and conflicts. *Environment, Development and Sustainability,**26*, 15681–15710. 10.1007/s10668-023-03270-5

[CR10] Coronado, E., Zepeda-Domínguez, J. A., Espinoza-Tenorio, A., Santamaria, D. C., Ramos-Muñoz, D., & Monzón-Alvarado, C. (2024). Institutional mapping and implementation of the sustainable development goals across co-existing industries: The case of the fisheries-oil system in Mexico. *The Extractive Industries and Society,**17*, Article 101390. 10.1016/J.EXIS.2023.101390

[CR11] Crespo-Guerrero, J. M., Jiménez-Pelcastre, A., & Nava-Martínez, J. D. (2019). Tensiones y conflictos territoriales en la pesca ribereña del Estado de Campeche, México (2013-2018). *Boletín De La Asociación De Geógrafos Españoles,**82*(2764), 1–53. 10.21138/bage.2764

[CR12] Cruz-Ramírez, C. J., Chávez, V., Silva, R., Muñoz-Perez, J. J., & Rivera-Arriaga, E. (2024). Coastal management: A review of key elements for vulnerability assessment. *Journal of Marine Science and Engineering,**12*, Article 386. 10.3390/jmse12030386

[CR13] Cuevas, E., Liceaga-Correa, M. A., & Uribe-Martínez, A. (2019). Ecological vulnerability of two sea turtle species in the Gulf of Mexico: An integrated spatial approach. *Endangered Species Research,**40*, 337–356. 10.3354/esr00984

[CR14] Diario Oficial de la Federación (DOF). (2012). Acuerdo por el que se expide la parte marina del Programa de Ordenamiento Ecológico Marino y Regional del Golfo de México y Mar Caribe y se da a conocer la parte regional del propio Programa (Continúa en la Segunda Sección). Retrieved August 23, 2025, from https://dof.gob.mx/nota_detalle.php?codigo=5279084&fecha=24/11/2012#gsc.tab=0

[CR15] Diario Oficial de la Federación (DOF). (2015). NORMA Oficial Mexicana NOM-062-SAG/PESC-2014, Para la utilización del Sistema de Localización y Monitoreo Satelital de Embarcaciones Pesqueras. Retrieved August 23, 2025, from https://dof.gob.mx/nota_to_doc.php%3Fcodnota%3D5399371

[CR16] Diario Oficial de la Federación (DOF). (2019). Acuerdo por el cual se establecen medidas para incrementar la seguridad y protección de las instalaciones petroleras marinas de la Sonda de Campeche. Retrieved August 23, 2025, from https://www.dof.gob.mx/nota_detalle.php?codigo=5570263&fecha=23/08/2019#gsc.tab=0

[CR17] Diario Oficial de la Federación (DOF). (2023). Acuerdo Secretarial Núm. 512/2023 por el que se expide la versión abreviada del Plan Nacional de Contingencia para Derrames de Hidrocarburos y Sustancias Nocivas Potencialmente Peligrosas en las Zonas Marinas Mexicanas. Retrieved August 23, 2025, from https://sidof.segob.gob.mx/notas/5712727

[CR18] Diario Oficial de la Federación (DOF). (2025). Acuerdo General CNH.E.08.01/2025 por el que la Comisión Nacional de Hidrocarburos declara la suspensión de los plazos y términos para la recepción, sustanciación y resolución de los actos, trámites y procedimientos sustanciados en la Comisión Nacional de Hidrocarburos, así como la suspensión de los periodos de exploración, evaluación de los contratos de exploración y extracción de hidrocarburos y los programas de transición. Retrieved April 3, 2026, from https://sidof.segob.gob.mx/notas/5748800

[CR19] Drakopulos, L., Silver, J. J., Nost, E., Gray, N., & Hawkins, R. (2023). Making global oceans governance in/visible with Smart Earth: The case of Global Fishing Watch. *Environment and Planning E: Nature and Space,**6*(2), 1098–1113. 10.1177/25148486221111786

[CR20] Eidsvig, U. M., Medina-Cetina, Z., Kveldsvik, V., Glimsdal, S., Harbitz, C. B., & Sandersen, F. (2011). Risk assessment of a tsunamigenic rockslide at Åknes. *Natural Hazards,**56*, 529–545.

[CR21] Elayam, M. M., Kerhoas, G., Lambert de Cursay, V., Ray, C., & Ménard, A. (2022). On the Interest of Hexagonal Abstraction of Maritime Information. In *OCEANS 2022*, Hampton Roads (pp. 1–6). IEEE. 10.1109/OCEANS47191.2022.9977062

[CR22] Ferrari, L., Flores-Hernández, J. R., & Hernández-Martínez, D. (2024). A 20 años del pico del petróleo en México: Análisis del sector hidrocarburos e implicaciones para el futuro energético nacional. *Revista Mexicana de Ciencias Geológicas,**41*(1), 66–86. 10.22201/cgeo.20072902e.2024.1.1770

[CR23] Füssel, H. M., & Klein, R. J. T. (2006). Climate change vulnerability assessments: an evolution of conceptual thinking. *Climate Change, 75*, 301–329.

[CR24] García-Cuéllar, J., Arreguín-Sánchez, F., Hernández-Vázquez, S., & Lluch-Cota, D. (2004). Impacto ecológico de la industria petrolera en la sonda de Campeche, México, tras tres décadas de actividad: Una revision. *Interciencia,**29*, 311–319.

[CR25] Ge, J., Polhill, J. G., & Craig, T. P. (2018). Too much of a good thing? Using a spatial agent-based model to evaluate “unconventional” workplace sharing programmes. *Journal of Transporte Geography,**69*, 83–97. 10.1016/j.jtrangeo.2018.04.005

[CR26] Gibbs, M. T., & Browman, H. I. (2015). Risk assessment and risk management: A primer for marine scientists. *ICES Journal of Marine Science,**72*(3), 992–996. 10.1093/icesjms/fsu232

[CR27] Global Fishing Watch, Inc. Copyright. (2026). Global Fishing Watch Mapper. Accessed on [August 2025]. [https://globalfishingwatch.org/map/]

[CR28] Grant, J. P. (1978). The conflict between the fishing and the oil industries in the North Sea: A case study. *Ocean & Coastal Management,**4*(2–4), 137–149.

[CR29] Helle, I., Mäkinen, J., Nevalainen, M., Afenyo, M., & Vanhatalo, J. (2020). Impacts of oil spills on Arctic marine ecosystems: A quantitative and probabilistic risk assessment perspective. *Environmental Science & Technology,**54*, 2112–2121. 10.1021/acs.est.9b07086 31971780 10.1021/acs.est.9b07086 PMC7145341

[CR30] Holsman, K., Samhouri, J., Cook, G., Hazen, E., Olsen, E., Dillard, M., Kasperski, S., Gaichas, S., Kelble, C. R., Fogarty, M., & Andrews, K. (2017). An ecosystem-based approach to marine risk assessment. *Ecosystem Health and Sustainability,**3*(1), Article e01256. 10.1002/ehs2.1256

[CR31] Hsu, F. C., Elvidge, C. D., Baugh, K., Zhizhin, M., Ghosh, T., Kroodsma, D., Susanto, A., Budy, W., Riyanto, M., Nurzeha, R., & Sudarja, Y. (2019). Cross-matching VIIRS boat detections with vessel monitoring system tracks in Indonesia. *Remote Sensing,**11*, Article 995. 10.3390/rs11090995

[CR32] Jiang, Z. P., Yuan, J., Hartman, S. E., & Fan, W. (2019). Enhancing the observing capacity for the surface ocean by the use of volunteer observing ship. *Acta Oceanologica Sinica,**38*(7), 114–120. 10.1007/s13131-019-1463-3

[CR33] Kashubsky, M., & Morrison, A. (2013). Security of offshore oil and gas facilities: Exclusion zones and ships’ routeing. *Australian Journal of Maritime & Ocean Affairs,**5*(1), 1–10. 10.1080/18366503.2013.10815725

[CR34] Kaynia, A. M., Papathoma-Köhle, M., Neuhäuser, B., Ratzinger, K., Wenzel, H., & Medina-Cetina, Z. (2008). Probabilistic assessment of vulnerability to landslide: Application to the village of Liechtenstein, Baden-Württemberg, Germany. *Engineering Geology,**101*, 33–48. 10.1016/j.enggeo.2008.03.008

[CR35] Keijser, X., Ripken, M., Mayer, I., Warmelink, H., Abspoel, L., Fairgrieve, R., & Paris, C. (2018). Stakeholder engagement in maritime spatial planning: The efficacy of a serious game approach. *Water,**10*, Article 724. 10.3390/w10060724

[CR36] Kroodsma, D. A., Mayorga, J., Hochberg, T., Miller, N. A., Boerder, K., Ferretti, F., Wilson, A., et al. (2018). Tracking the global footprint of fisheries. *Science,**359*, 904–908. 10.1126/science.aao5646 29472481 10.1126/science.aao5646

[CR37] Kusters, J. E. H., van Kann, M. G., & Zuidema, C. (2025). Spatial conflict resolution in marine spatial plans and permitting procedures for offshore wind energy: An analysis of measures adopted in Denmark, England and the Netherlands. *Frontiers in Marine Science,**12*, Article 1468734. 10.3389/fmars.2025.1468734

[CR38] Lara-Mendoza, R. E., Vásquez-Ortíz, M., Caña-Hernández, S., Zarate-Herrera, M. T. (2026). Small-Scale Fisheries and Oil Marine Infrastructure Interactions: Challenges for Marine Sustainability and Territorial Planning in the Southern Gulf of Mexico. In: Espinoza-Tenorio, A., Ramos-Muñoz, D. E., Núñez-Lara, E. (eds) The Fishing and Oil Industries in Coexistence. Springer, Cham. 10.1007/978-3-032-02621-7_17

[CR39] Li, Y., Umair, M., Guliyeva, S., & Shakaraliyeva, Z. (2025). The extractive industries transparency initiative: Achieving disclosure, but falling short on corruption reduction. *The Extractive Industries and Society,**22*, Article 101602. 10.1016/J.EXIS.2024.101602

[CR40] Liceaga-Correa, M. A., Uribe-Martínez, A., & Cuevas, E. (2021). Ecological vulnerability of adult female marine turtles as indicators of opportunities for regional socioecosystem management in the southern Gulf of Mexico. *Sustainability, 14*, 184. 10.3390/su14010184

[CR41] Lindkvist, E., Wijermans, N., Daw, T. M., González-Mon, B., Giron-Nava, A., Johnson, A. F., van Putten, I., et al. (2020). Navigating complexities: Agent-based modeling to support research, governance, and management in small-scale fisheries. *Frontiers in Marine Science,**6*, Article 733. 10.3389/fmars.2019.00733

[CR42] López-Rocha, J. A., Ramos-Miranda, J., Velázquez-Abunader, I., Cabrera, M. A., Salas, S., & Flores-Hernández, D. (2021). *Artes y Métodos de Pesca de la península de Yucatán. Universidad Autónoma de Campeche*. Centro de Investigación y de Estudios Avanzados del IPN Unidad Mérida - Universidad Nacional Autónoma de México. México. 10.26359/EPOMEX0121

[CR43] Montejo-Damián, K. C., Díaz-Perera, M., & Espinoza-Tenorio, A. (2022). The social construction of risk: A local perspective of the vulnerability of artisanal fisheries to climate change. *Coastal Studies and Society,**1*(1), 55–77. 10.1177/26349817221080864

[CR44] Müller, B., Bohn, F., Drebler, G., Groeneveld, J., Klassert, C., Martin, R., Schlüter, M., et al. (2013). Describing human decisions in agent-based models – ODD + D, an extension of the ODD protocol. *Environmental Modelling & Software,**48*, 37–48. 10.1016/j.envsoft.2013.06.003

[CR45] Nava-Fuentes, J. C., Arenas-Granados, P., & Cardoso-Martins, F. (2018). Integrated coastal management in Campeche, Mexico; a review after the Mexican marine and coastal national policy. *Ocean & Coastal Management,**154*, 34–45.

[CR46] Nelson, J. R., Grubesic, T. H., Sim, L., & Graham, J. (2015). Approach for assessing coastal vulnerability to oil spills for prevention and readiness using GIS and the Blowout and Spill Occurrence Model. *Ocean and Coastal Management,**112*, 1–11. 10.1016/j.ocecoaman.2015.04.014

[CR47] Office for Coastal Management. (2024) Nationwide Automatic Identification System 2021. Retrieved August 23, 2025, from https://www.fisheries.noaa.gov/inport/item/65082.

[CR48] Oliveto-Andrade, A., Espinoza-Tenorio, A., Ramos-Muñoz, D., & Pérez-Jiménez, J. C. (2024). Understanding the motivations of young people from marginalized rural communities to participate in small-scale fisheries in oil territories of the Gulf of Mexico. *Ocean & Coastal Management,**248*, Article 106947. 10.1016/j.ocecoaman.2023.106947

[CR49] Paolo, F., Kroodsma, D., Raynor, J., Hochberg, T., Davis, P., Cleary, J., Marsaglia, L., Orofino, S., Thomas, C., & Halpin, P. (2024). Satellite mapping reveals extensive industrial activity at sea. *Nature,**625*, 85–91. 10.1038/s41586-023-06825-8 38172362 10.1038/s41586-023-06825-8 PMC10764273

[CR50] Park, J., Van Osdel, J., Turner, J., Farthing, C. M., Miller, N. A., Linder, H. L., Ortuño-Crespo, G., Camine, G., & Kroodsma, D. A. (2023). Tracking elusive and shifting identities of the global fishing fleet. *Science,**9*, Article eabp8200. 10.1126/sciadv.abp8200 10.1126/sciadv.abp8200 PMC9848426 36652516

[CR51] Pascoe, S., & Innes, J. P. (2018). Economic impacts of the development of an offshore oil and gas industry on fishing industries: A review of experiences and assessment methods. *Reviews in Fisheries Science & Aquaculture,**26*, 350–370. 10.1080/23308249.2018.1436521

[CR52] Pavlov, V., Martins de Aguiar, V. C., Hole, L. R., & Pongrácz, E. (2022). A 30-year probability map for oil spill trajectories in the Barents Sea to assess potential environmental and socio-economic threats. *Resources,**11*, Article 1. 10.3390/resources11010001

[CR53] PEMEX. (2023). *Informe de Sostenibilidad*. Petróleos Mexicanos, Ciudad de México, México https://www.pemex.com/etica_y_transparencia/transparencia/informes/Documents/informe_sostenibilidad_2023_esp.pdf. Accessed 15 Feb 2026

[CR54] Pirasteh, S., Fang, Y., Mafi-Gholami, D., Abulibdeh, A., Nouri-Kamari, A., & Khonsari, N. (2024). Enhancing vulnerability assessment through spatially explicit modeling of mountain social-ecological systems exposed to multiple environmental hazards. *Science of the Total Environment,**930*, Article 172744. 10.1016/j.scitotenv.2024.172744 38685429 10.1016/j.scitotenv.2024.172744

[CR55] Python Software Foundation. (2024). *Python language reference*, (version 3.12.) [computer software). https://www.python.org. Accessed 1 Nov 2023.

[CR56] QGIS.org. (2025). QGIS Geographic Information System. QGIS Association.Retrieved August 23, 3035, from http://www.qgis.org

[CR57] Quijano-Quiñones, D. R., López-Rocha, J. A., Hernández-Herrera, I., & Torres-Irineo, E. (2021). Spatial dynamics modeling of small-scale fishing fleets with a random walk approach. *Frontiers in Marine Science,**8*, Article 669112. 10.3389/fmars.2021.669112

[CR58] Quijano, D., Salas, S., Monroy-García, C., Dreyfus-León, M., & Torrres-Irineo, E. (2023). Identifying fisheries operations in tropical multispecies fisheries: A comparative analysis of multivariate approaches and neural networks. *Fisheries Research,**263*, Article 106692.

[CR59] Quist, L. M., & Nygren, A. (2015). Contested claims over space and identity between fishers and the oil industry in Mexico. *Geoforum,**63*, 44–54. 10.1016/j.geoforum.2015.05.015

[CR60] Ramos-Muñoz, D. E., Ramos-Reyes, R., Zamora-Cornelio, L. F., Hernández-De la Cruz, A., & Espinoza-Tenorio, A. (2019). Exclusión en el Golfo de México: Una visión desde los pescadores sobre la industria petrolera en Tabasco. *Cuadernos de Geografía: Revista Colombiana de Geografía,**28*(2), 357–372. 10.15446/rcdg.v28n2.73511

[CR61] Rodríguez-García, H. I., Ramos-Muñoz, D., & Ramírez-Pacheco, A. A. (2022). Infrastructure and territorial transformations in Tabasco, Mexico (1950-2017): An approach from social cartography. *Economía, Sociedad y Territorio,**XXII*(69), 571–601. 10.22136/est20221787

[CR62] Rodriguez-Viñas, J., Ortega-Fernandez, I., & Sotos Martínez, E. (2023). Hexanonymity: A scalable geo-positioned data clustering algorithm for anonymisation purposes. *IEEE European Symposium on Security and Privacy Workshops (EuroS&PW)*. 10.1109/EuroSPW59978.2023.00050

[CR63] Saaty, T. L. (2008). Decision making with the analytic hierarchy process. *International Journal of Services Sciences,**1*, 83–98. 10.1504/IJSSCI.2008.017590

[CR64] Salazar-De-la-Cruz, C. C., Zepeda-Domínguez, J. A., Espinoza-Tenorio, A., & Ramos-Muñoz, D. E. (2020). Governance networks in marine spaces where fisheries and oil coexist: Tabasco, México. *The Extractive Industries and Society,**7*, 676–685. 10.1016/j.exis.2020.03.012

[CR65] Saldaña-Ruíz, L. E., Pérez-Brunius, P., Sosa-Nishizaki, O., Romo-Curiel, A. E., Ramírez-Mendoza, Z., Fajardo-Yamamoto, A., García-Aguilar, M. C., & Ramírez-León, M. R. (2020). Vulnerabilidad del ecosistema marino del sur del golfo de México y el mar Caribe mexicano a derrames de petróleo. In P. Pérez Brunius & M. L. Aguirre-Macedo (Eds.), *Vulnerabilidad ecológica del golfo de México ante derrames a gran escala (tomo II)* (1st ed., pp. 17–41). CICESE, CINVESTAV, UNAM.

[CR66] Sameoto, J. A., Brown, C. J., Keith, D. M., & McGonigle, C. (2025). Accuracy of calculated speeds from vessel monitoring system data: Considerations for fisheries management. *North American Journal of Fisheries Management,**45*, 1190–1203. 10.1093/najfmt/vqaf096

[CR67] Sosa-Nishizaki, O., Romo-Curiel, A. E. :, Ramírez-Mendoza, Z., Fajardo-Yamamoto, A., García-Aguilar, M. C., & Ramírez-León, M. R. (2020). Evaluación de la vulnerabilidad de los peces pelágicos ante escenarios de derrame de petróleo profundos en el golfo de México. In P. Pérez Brunius & M. L. Aguirre-Macedo (Eds.), *Vulnerabilidad ecológica del golfo de México ante derrames a gran escala (tomo II)* (1st ed., pp. 17–41). CICESE, CINVESTAV, UNAM.

[CR68] Stelzenmüller, V., Ellis, J. R., & Rogers, S. I. (2010). Towards a spatially explicit risk assessment for marine management: Assessing the vulnerability of fish to aggregate extraction. *Biological Conservation,**143*, 230–238. 10.1016/j.biocon.2009.10.007

[CR69] Stelzenmüller, V., Fock, H. O., Gimpel, A., Rambo, H., Diekmann, R., Probst, W. N., Callies, U., Bockelmann, F., Neumann, H., & Kröncke, I. (2015). Quantitative environmental risk assessments in the context of marine spatial management: Current approaches and some perspectives. *ICES Journal of Marine Science,**72*(3), 1022–1042. 10.1093/icesjms/fsu206

[CR70] Sun, S., Hu, C., & Tunnell, J. W., Jr. (2015). Surface oil footprint and trajectory of the Ixtoc-I oil spill determined from Landsat/MSS and CZCS observations. *Marine Pollution Bulletin,**101*, 632–641. 10.1016/j.marpolbul.2015.10.036 10.1016/j.marpolbul.2015.10.036 26507512

[CR71] Temitope-Yekeen, S., & Balogun, A. L. (2020). Advances in remote sensing technology, machine learning and deep learning for marine oil spill detection, prediction and vulnerability assessment. *Remote Sensing,**12*, Article 3416. 10.3390/rs12203416

[CR72] Torres-Irineo, E., Salas, S., Euán-Ávila, J. I., Palomo, L. E., Quijano-Quiñones, D. R., Coronado, E., & Joo, R. (2021). Spatio-temporal determination of small-scale vessels’ fishing grounds using a vessel monitoring system in the southeastern Gulf of Mexico. *Frontiers in Marine Science,**8*, Article 643318. 10.3389/fmars.2021.643318

[CR73] Trygg, K., & Wenander, H. (2022). Strategic spatial planning for sustainable development – Swedish planners’ institutional capacity. *European Planning Studies,**30*, 1985–2001. 10.1080/09654313.2021.2001792

[CR74] Tyack, P. L., Thomas, L., Costa, D. P., Hall, A. J., Harris, C. M., Harwood, J., Kraus, S. D., Miller, P. J. O., Moore, M., Photopoulou, T., Pirotta, E., Rolland, R. M., Schwacke, L. H., Simmons, S. E., & Southall, B. L. (2022). Managing the effects of multiple stressors on wildlife populations in their ecosystems: Developing a cumulative risk approach. *Proceedings of the Royal Society B: Biological Sciences,**289*, Article 20222058. 10.1098/rspb.2022.2058 10.1098/rspb.2022.2058 PMC9709579 36448280

[CR75] United Nations Office for Disaster Risk Reduction. (2025). *Global assessment report on disaster risk reduction 2025: Resilience pays: Financing and investing for our future*. Geneva. https://www.undrr.org/gar. Accessed 15 Jun 2026

[CR76] Uribe-Martínez, A., Cruz-Pech, J., Velasco, J., Trujillo-Córdova, J., & Cuevas, E. (2020). Evaluación espaciotemporal de hidrocarburos en la superficie marina por derrames y por emisiones naturales en la Sonda de Campeche [Spatiotemporal assessment of hydrocarbons on the marine surface by spills and by natural emissions in the Campeche Sound]. Reunión Anual de la Unión de Geofísica Mexicana, November 2–6, 2020 (Virtual). Retrieved August 23, 2025, from https://geos.cicese.mx/index.php/geos/issue/view/25

[CR77] Uribe-Martínez, A., Espinoza-Tenorio, A., Cruz-Pech, J. B., Cupido-Santamaría, D. G., Trujillo-Córdova, J. A., García-Nava, H., Flores-Vidal, X., Gudiño-Elizondo, N., Herguera, J. C., Appendini, C. M., & Cuevas, E. (2024). An affordable operational oil spill monitoring system in action: A diachronic multiplatform analysis of recent incidents in the southern Gulf of Mexico. *Environmental Monitoring and Assessment,**196*, Article 1069. 10.1007/s10661-024-13161-5 39419911 10.1007/s10661-024-13161-5 PMC11486827

[CR78] Uribe-Sandoval, L.M. (2025). *Uso del espacio pesquero de una cooperativa artesanal y zonas de presencia recurrente de petróleo en la Sonda de Campeche*. Degree Dissertation. Escuela Nacional de Estudios Superiores, Unidad Morelia. Universidad Nacional Autónoma de México. 86 p.

[CR79] Wakida-Kusunoki, A. T., & Caballero-Chavez, V. (2009). Effects of the Kab 121 well oil spill on artisanal fishing in the coast of Campeche and Tabasco, Mexico. *Ciencia Pesquera, Mayo,**17*, 12.

[CR80] Wakida-Kusunoki, A. T., Arreguín-Sánchez, F., González-Cruz, A., & Ponce-Palafox, J. T. (2010). Análisis de la distribución espacial del esfuerzo pesquero de la flota camaronera mexicana en el Golfo de México y el mar Caribe por medio del sistema satelital de monitoreo de embarcaciones. *Ciencia Pesquera,**18*(1), 43–50.

[CR81] Witt, M. J., & Godley, B. J. (2007). A step towards seascape scale conservation: Using vessel monitoring systems (VMS) to map fishing activity. *PLoS One,**2*(10), Article e1111. 10.1371/journal.pone.0001111 17971874 10.1371/journal.pone.0001111 PMC2040201

[CR82] Zacharias, M. A., & Gregr, E. J. (2005). Sensitivity and vulnerability in marine environments: An approach to identifying vulnerable marine areas. *Conservation Biology,**19*, 86–97. 10.1111/j.1523-1739.2005.00148.x

[CR83] Zalik, A. (2009). Zones of exclusion: Offshore extraction, the contestation of space and physical displacement in the Nigerian Delta and the Mexican Gulf. *Antipode,**41*(3), 557–582. 10.1111/j.1467-8330.2009.00687.x

