# Supplementary material for: Vulnerability and risk assessment of the coexistence of oil and fishing industries in the Southern Gulf of Mexico
Source: Environ Monit Assess. 2026 Jun 24;198(7):762. doi: 10.1007/s10661-026-15583-9 (PMC13294270; doi:10.1007/s10661-026-15583-9)
Supplement: Supplementary file 1 — (556 KB DOCX) [file 10661_2026_15583_MOESM1_ESM.docx]

**Spatial Coexistence of Oil and Fishing Industries in the Southern Gulf of Mexico: Assessing Vulnerability and Risk of Industry Interactions on Artisanal and Industrial Fisheries**

**Supplementary Materials**


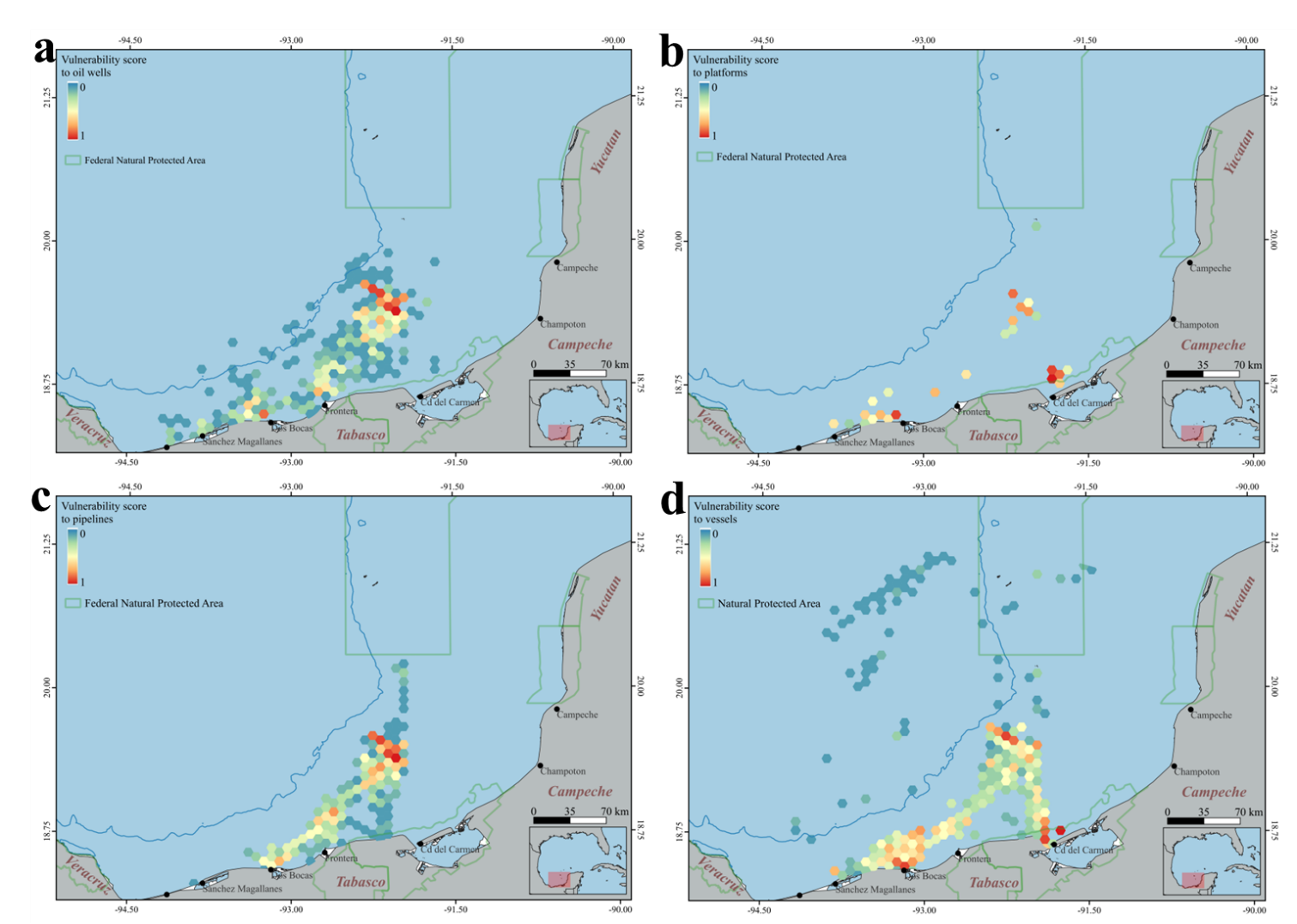


Figure S1. Individual vulnerabilities to the four elements of oil industry evaluated in this study: (a) wells, (b) platforms, (c) pipelines, and (d) vessels.
